# The BILAG2004-Pregnancy Index is a valid disease activity outcome measure for pregnant SLE patients

**DOI:** 10.1093/rap/rkac081

**Published:** 2022-10-03

**Authors:** Chee-Seng Yee, Munther Khamashta, Mohammed Akil, Rachael Kilding, Ian Giles, David Williams, Ian N Bruce, Caroline Gordon

**Affiliations:** Department of Rheumatology, Doncaster and Bassetlaw Teaching Hospitals NHS Foundation Trust, Doncaster, UK; Department of Women and Children’s Health, King’s College London, London, UK; Department of Rheumatology, Sheffield Teaching Hospitals NHS Trust, Sheffield, UK; Department of Rheumatology, Sheffield Teaching Hospitals NHS Trust, Sheffield, UK; Centre for Rheumatology Research, University College London, London, UK; Department of Obstetrics, University College London Hospitals NHS Foundation Trust, London, UK; Centre for Epidemiology Versus Arthritis, University of Manchester, Manchester, UK; Rheumatology Research Group, University of Birmingham, Birmingham, UK

**Keywords:** BILAG2004-pregnancy, SLE, disease activity, pregnancy, construct validity, criterion validity, sensitivity to change

## Abstract

**Objectives:**

This study was to determine whether the BILAG2004-Pregnancy Index (BILAG2004-P) has construct/criterion validity and is sensitive to change.

**Methods:**

This was an observational multicentre study that recruited pregnant SLE patients. Data were collected on disease activity [using the BILAG2004-P and Physician Global Assessment (PGA)], investigations and therapy at each assessment. The overall BILAG2004-P score as determined by the highest score achieved by any system was used in the analysis. Cross-sectional analysis was used for construct and criterion validity. The comparison was with C3, C4 and anti-dsDNA for construct validity, while it was with change in therapy and PGA in criterion validity. Sensitivity to change was assessed by determining the relationship between the change in BILAG2004-P and the change in therapy between two consecutive visits.

**Results:**

A total of 97 patients with 112 pregnancies were recruited. There were 610 assessments available for construct/criterion validity analysis (98.2% of pregnancies had more than one assessment) and 497 observations for sensitivity to change analysis. Increasing BILAG2004-P scores were associated with low C3. The active BILAG2004-P score (grade A or B) was associated with an increase in therapy and the PGA of active disease. There was an increasing likelihood of higher overall scores with an increase in therapy and the PGA of active disease. In the sensitivity to change analysis, an increase in the BILAG2004-P score was associated with an increase in therapy and inversely associated with a decrease in therapy. A decrease in the BILAG2004-P score was associated with a decrease in therapy and inversely associated with an increase in therapy.

**Conclusion:**

The BILAG2004-P has criterion validity and is sensitive to change.

Key messages

- The BILAG2004-Pregnancy Index has criterion validity.
- The BILAG2004-Pregnancy Index is sensitive to change.
- The BILAG2004-Pregnancy Index is valid for assessment of SLE disease activity in pregnancy.

## Introduction

As SLE affects mainly women of childbearing age, more women with SLE are becoming or potentially are able to become pregnant with improvement in the management of SLE. However, many pathophysiological features of pregnancy may mimic manifestations of SLE disease activity and confound assessment of SLE disease activity during pregnancy. Therefore, standardized assessment of SLE disease activity outcome measures that are developed for the non-pregnant state need to be modified to take into account these changes before it can be used in pregnancy.

The BILAG2004-Pregnancy Index (BILAG2004-P) is a modification of the BILAG-2004 index for assessment of SLE disease activity in pregnancy [1]. The modifications were to take into account pathophysiological changes in pregnancy. Most of the changes were to the glossary, with major changes in the scoring of the renal system. These modifications have been described in detail previously [1]. The development of this index enables standardized assessment of SLE disease activity outcomes during pregnancy. As it has a similar structure to the BILAG-2004 index [2], with nine system scores, it allows for a seamless transition in reporting between pregnant and non-pregnant states. A previous study showed the BILAG2004-P to be reliable [1]. The case record form, glossary and scoring scheme of this index are available as supplementary material (Supplementary Data S1–S3, available at *Rheumatology Advances in Practice* online). This analysis reports on the construct validity, criterion validity and sensitivity to change of the BILAG2004-P.

## Methods

This was a multicentre study involving five centres in the UK that recruited SLE patients who were pregnant and fulfilled the revised ACR criteria for classification of SLE [3]. These five centres (Manchester, Birmingham, University College London, St Thomas’ Hospital and Sheffield) had dedicated clinics looking after pregnant patients with rheumatic diseases. Patients were excluded from the study if they were <18 years of age or were unable to provide valid consent. This study was approved by the Trent Research Ethics Committee. It was carried out in accordance with the Helsinki Declaration and written informed consent was obtained from patients.

At each assessment, data were collected on disease activity [with BILAG2004-P and Physician Global Assessment (PGA)], investigations and therapy. The BILAG2004-P is a system-based index with nine ordinal system scores from grade A, indicating high disease activity, to grade E, indicating no disease activity and the system was not previously affected [1]. The overall BILAG2004-P score as determined by the highest score achieved by any individual system was used in the analysis. Grades D and E were combined for the analysis, as both indicate inactivity. A robust definition for change in treatment was used in the analysis that was similar to the one used in validation of the BILAG-2004 index (see Supplementary Data S4, available at *Rheumatology Advances in Practice* online) [2, 4].

All analyses were performed using Stata for Windows version 8 (StataCorp, College Station, TX, USA). Statistical analysis was with logistic regression and the robust estimator of variance was used to allow for correlation between assessments from the same patient. Results were reported as odds ratios (ORs) with 95% CIs.

### Construct and criterion validity

This was a cross-sectional analysis. For construct validity, the overall BILAG2004-P score was compared with constructs that were C3, C4 and anti-dsDNA levels. The levels of C3, C4 and anti-dsDNA (by ELISA) were measured locally as part of routine practice using standard methodology by accredited laboratories. In criterion validity, the criterion for active disease used for comparison with the overall BILAG2004-P score were change in therapy and the PGA of active disease. The PGA of active disease was a yes/no response to the question ‘Did this patient have active SLE disease?’. Change in therapy was classified into two categories: an increase in therapy and no increase in therapy. Although the scoring of the index was based on the physician’s intention to treat, change in treatment had no bearing on the score of the index. Sensitivity, specificity, positive predictive value (PPV) and negative predictive value (NPV) were calculated for criterion validity analysis.

### Sensitivity to change

In this longitudinal analysis, external responsiveness was used to assess the extent to which changes in the overall BILAG2004-P score over time relate to a corresponding change in therapy between two consecutive assessments [5]. Therefore, each observation for the analysis was derived from the changes that occurred between two consecutive assessments. There were three categories of change in therapy: no change, an increase in therapy and a decrease in therapy. Maximum-likelihood multinomial and binary logistic regression were used with change in therapy as the outcome variable and changes in the overall BILAG2004-P score as the explanatory variable. The baseline comparator for change in overall BILAG2004-P score was ‘minimal change in score’ as defined by ‘no change in score’ and change from grade D/E to C. The ability of change in overall BILAG2004-P score to predict a change in therapy was also assessed with the calculation of sensitivity, specificity, PPV and NPV.

## Results

A total 97 pregnant SLE patients were recruited, who contributed 112 pregnancies. The ethnicity of the patients was 57.7% Caucasian, 18.6% South Asian, 17.5% African-Caribbean and 6.1% others. At the time of entry into the study for each pregnancy, the mean age was 31.3 years (s.d. 5.4) and the mean disease duration was 7.4 years (s.d. 5.1). This is summarized in Table 1.

**Table 1.** Baseline demographics of recruited patients (112 pregnancies from 97 patients)

Patient characteristics	Values
Ethnicity (*n* = 97 patients), %	
Caucasian	57.7
South Asian	18.6
African-Caribbean	17.5
Other	6.1
Age at recruitment (*n* = 112 pregnancies), years, mean (s.d.)	31.3 (5.4)
Disease duration at recruitment (*n* = 112 pregnancies), years, mean (s.d.)	7.4 (5.1)

### Construct and criterion validity

There were 610 assessments available for this cross-sectional analysis (98.2% of pregnancies had more than one assessment). A total of 15.7% of assessments had active disease scores by the BILAG2004-P with overall scores of grade A or B and 21.5% of assessments were deemed to be active by the PGA. The distribution of disease activity across the systems is summarized in Table 2. The prevalence of low C3, low C4 and elevated anti-dsDNA were 12.2% (of 510 assessments), 21.8% (of 515 assessments) and 22.0% (of 505 assessments), respectively. There was an increase in therapy in 11.6% of assessments while a decrease in therapy occurred in 7% of assessments.

**Table 2.** Distribution of disease activity across systems according to the BILAG2004-P (*n* = 610 assessments)

BILAG2004-P systems	BILAG2004-P grade, *n* (%)			
	A	B	C	D/E
Constitutional	0	0	9 (1.5)	601 (98.5)
Mucocutaneous	4 (0.7)	54 (8.9)	96 (15.7)	456 (74.8)
Neuropsychiatric	1 (0.2)	1 (0.2)	0	608 (99.7)
Musculoskeletal	2 (0.3)	19 (3.1)	143 (23.4)	446 (73.1)
Cardiorespiratory	0	5 (0.8)	5 (0.8)	600 (98.4)
Gastrointestinal	0	0	0	610 (100)
Ophthalmic	0	0	0	610 (100)
Renal	3 (0.5)	11 (1.8)	5 (0.8)	591 (96.9)
Haematological	1 (0.2)	4 (0.7)	204 (33.4)	401 (65.7)

Analysis with ordinal logistic regression showed that there was a significant association between increasing overall BILAG2004-P score with low C3 [OR 2.0 (95% CI 1.1, 3.5)] but not with low C4 [OR 1.0 (95% CI 0.6, 1.9)] or elevated anti-dsDNA [OR 1.2 (95% CI 0.7, 2.1)].

The active overall BILAG2004-P score (grade A or B) was significantly associated with an increase in therapy [OR 18.0 (95% CI 10.3, 31.5)] and the PGA of active disease [OR 103.0 (95% CI 33.0, 322.2)]. There was an increasing likelihood of a higher overall score with an increase in therapy and the PGA of active disease (Table 3). There was total agreement between a grade A score and the PGA of active disease. Table 4 summarizes the ability of the active overall BILAG2004-P score in predicting an increase in therapy and the PGA of active disease. There was good specificity and NPV.

**Table 3.** Logistic regression analysis comparing overall BILAG2004-P scores with an increase in therapy and the PGA of active disease

Overall BILAG2004-P score	Increase in therapy, OR (95% CI)	PGA active disease, OR (95% CI)
D/E (reference)	–	–
C	4.2 (1.3, 12.8)	37.7 (4.8, 292.6)
B	43.7 (14.1, 135.6)	1849.3 (192.1, 17 800.8)
A	144.0 (35.5, 584.3)	∞

**Table 4.** Ability of active disease by overall BILAG2004-P score to predict an increase in therapy and the PGA of active disease

Calculation	Increase in therapy, % (95% CI)	PGA active disease, % (95% CI)
Sensitivity	64.8 (51.8, 75.9)	66.4 (55.2, 76.0)
Specificity	90.7 (86.5, 93.7)	98.1 (94.8, 99.3)
PPV	47.9 (37.6, 58.4)	90.6 (76.5, 96.6)
NPV	95.1 (92.8, 96.7)	91.4 (87.2, 94.3)

BILAG2004-P: BILAG2004-Pregnancy Index; PGA: Physician’s Global Assessment.

### Sensitivity to change

There were 497 observations available for this longitudinal analysis. An increase in therapy was observed in 11.7% of observations and a decrease in therapy occurred in 11.7% of observations. The distribution of changes in the overall BILAG2004-P score is summarized in Table 5.

**Table 5.** Cross-tabulation of changes in the overall BILAG2004-P score against the change in therapy longitudinally (*n* = 497 observations)

Change in overall BILAG2004-P score	Change in therapy, *n* (%)		
	Decrease	No change	Increase
Minimal change	36 (7.2)	285 (57.3)	33 (6.6)
Decrease to C/D	20 (4.0)	79 (15.9)	1 (0.2)
Decrease to B	2 (0.4)	2 (0.4)	1 (0.2)
Increase to B	0	14 (2.8)	18 (3.6)
B to A	0	1 (0.2)	1 (0.2)
C/D to A	0	0	4 (0.8)

Multinomial logistic regression analysis showed that an increase in the overall BILAG2004-P score was significantly associated with an increase in therapy [OR 13.2 (95% CI 5.8, 30.1)] and was inversely associated with a decrease in therapy [OR <0.1 (95% CI <0.1, <0.1)]. On the other hand, a decrease in the overall BILAG2004-P score was significantly associated with a decrease in therapy [OR 2.2 (95% CI 1.3, 3.7)] and was inversely associated with an increase in therapy [OR 0.2 (95% CI <0.1, 0.96)]. An increase in therapy was unlikely in the absence of an increase in the overall BILAG2004-P score, as evident by the excellent specificity and NPV (Table 6).

**Table 6.** Ability of increase in the overall BILAG2004-P score to predict an increase in therapy

Calculation	% (95% CI)
Sensitivity	39.7 (4.4)
Specificity	96.6 (94.6, 97.9)
PPV	40.0 (30.0, 51.0)
NPV	93.8 (90.6, 95.9)

## Discussion

This was a relatively large multicentre study assessing the construct validity, criterion validity and sensitivity to change of the BILAG2004-P. However, most of the assessments had low-level disease activity (grade C, D or E scores), with >75% of assessments (for construct and criterion validity analysis) and observations (for sensitivity to change analysis) having no change in therapy. Despite this, we demonstrated that the BILAG2004-P has criterion validity from its significant association with both an increase in therapy and the PGA of active disease. The longitudinal analysis showed that it is sensitive to change through the relationship of change in the index score with change in therapy. In the analysis of construct validity, the only significant association demonstrated was with low C3.

The major limitation of this study is the low prevalence of active disease as noted in the small numbers of grade A and B scores. Only 2.3% of the assessments had active renal disease (grade A or B), which was shown to have the strongest association with low C3/C4 and elevated anti-dsDNA [6–8]. It is to be expected that most pregnant SLE patients would have minimal disease activity, as current management of SLE patients is to minimize the risk of active disease before and during pregnancy in order to improve pregnancy outcome [9]. This may explain the inability to demonstrate the association between active disease scores and low C4 or elevated anti-dsDNA.

In addition, most of the disease activity affected the mucocutaneous, musculoskeletal and renal systems, thus it is not possible to assess the index through its full range of disease activity with this dataset. Despite this, we do not anticipate any issues with regards to the validity of this index, as this index was modified (specifically for use in pregnancy) from the BILAG-2004 index, which was comprehensively validated [2, 4, 10]. As such, it would be reasonable to conclude that the BILAG2004-P is valid for use to assess SLE disease activity during pregnancy.

In conclusion, the BILAG2004-P will be the only SLE disease activity index that has undergone a thorough validation process in pregnant SLE patients, as it has been shown to be reliable previously [1], and this study has demonstrated construct/criterion validity along with sensitivity to change. There are other disease activity indices that have been used to assess SLE disease activity during pregnancy, such as the SLE-DAS [11] and SLEPDAI [12], but they have not been validated for use in pregnancy. Unlike the BILAG2004-P, which is a system-based disease activity index, these other indices are global disease activity indices that summarize disease activity into a single global score. The majority of these other indices are based on modifications of existing indices that were developed for non-pregnant states to take into account pathophysiological changes in pregnancy. These other indices have been discussed succinctly in a review article [12].

## Supplementary data

Supplementary data are available at *Rheumatology Advances in Practice* online.

## Supplementary Material

rkac081_Supplementary_Data

## Data Availability

The data underlying this article will be shared upon reasonable request to the corresponding author.
