# Supplementary material for: The BILAG2004-Pregnancy Index is a valid disease activity outcome measure for pregnant SLE patients
Source: Rheumatol Adv Pract. 2022 Oct 3;6(3):rkac081. doi: 10.1093/rap/rkac081 (PMC9585949; doi:10.1093/rap/rkac081)
Supplement: rkac081_Supplementary_Data [file rkac081_supplementary_data.zip › Supplementary Data S4. Definition of Change in Therapy.docx]

**SUPPLEMENTARY MATERIAL**

**Definition of Change in Therapy**

This has been described previously (1–5)**.** The medications of interest were immunosuppressives, antimalarials, glucocorticoids, biological therapy, topical glucocorticoids, topical immunosuppressives, intravenous immunoglobulins, plasmapheresis and anticoagulation.

Increase in therapy was defined as any increase in the medications of interest regardless of any concomitant reduction in other medications. Decrease in therapy was defined as any reduction in any of the medication of interest without any concomitant increase in other medications. However, change in therapy was not just a simple change in the dose of the medications. The following special circumstances were taken into account:

1. Dosing levels based on body weight
2. Step-down change of immunosuppressive therapy
3. Gradual escalation of immunosuppressive therapy following initiation
4. Increase in immunosuppressive therapy for steroid-sparing effect
5. Initiation of anticoagulation for active SLE disease
6. Reduction or discontinuation of therapy due to side effects

For some immunosuppressives, different dosing levels based on body weight were considered in the definition of change in therapy (Table A). A change in therapy was deemed to have occurred when there had been a change in the dosing levels of these medications. For medications that were not listed in Table below, a simple change in dose would constitute a change in therapy.

Table A. Dosing levels of medications used for definition of change in therapy

| Medications | Level 1 | Level 2 | Level 3 |
| --- | --- | --- | --- |
| Azathioprine  Ciclosporin A  Tacrolimus | < 1 mg/kg/day  < 2 mg/kg/day  < 0.10 mg/kg/day | 1-2.4 mg/kg/day  2-3 mg/kg/day  0.10-0.15 mg/kg/day | ≥ 2.5 mg/kg/day  > 3 mg/kg/day  > 0.15 mg/kg/day |

If an immunosuppressive agent was started only for its steroid-sparing effect, this was not considered to be an increase in therapy. If anticoagulation was initiated for the reason of active disease (which was clarified with the local investigator) and in the presence of immunosuppressives or high dose steroids, this was considered to be an increase in therapy.

As most immunosuppressives have potential toxicity, it was common practice to start at a low dose and gradually escalated to the target dose. To take this into account, any increase in the dose of immunosuppressives within the first 3 months of initiation was considered to be part of an escalation plan to achieve the target dose and not as an increase in therapy. Similarly, it was common practice to reduce glucocorticoids dose gradually during this period as part of the escalation plan. Therefore, any concomitant reduction in glucocorticoids dose during escalation phase was not considered as a reduction in therapy.

If any medication was decreased or discontinued due to side effects, this was also not considered to be a reduction in therapy.

**References**

1. Yee CS, Farewell V, Isenberg DA, Griffiths B, Teh LS, Bruce IN, et al. The BILAG-2004 index is sensitive to change for assessment of SLE disease activity. Rheumatology. 2009;48(6):691–5.

2. Yee CS, Cresswell L, Farewell V, Rahman A, Teh LS, Griffiths B, et al. Numerical scoring for the BILAG-2004 index. Rheumatology. 2010;49(9):1665–9.

3. Yee CS, Farewell V, Isenberg DA, Rahman A, Teh LS, Griffiths B, et al. British Isles Lupus Assessment Group 2004 index is valid for assessment of disease activity in systemic lupus erythematosus. Arthritis Rheum. 2007;56(12):4113–9.

4. Yee CS, Gordon C, Isenberg DA, Griffiths B, Teh LS, Bruce IN, et al. The BILAG-2004 systems tally-a novel way of representing the BILAG-2004 index scores longitudinally. Rheumatol (United Kingdom). 2012;51(11):2099–105.

5. Yee CS, Farewell VT, Isenberg DA, Griffiths B, Teh LS, Bruce IN, et al. The use of systemic lupus erythematosus disease activity index-2000 to define active disease and minimal clinically meaningful change based on data from a large cohort of systemic lupus erythematosus patients. Rheumatology. 2011;50(5):982–8.
