# Supplementary material for: The BILAG2004-Pregnancy Index is a valid disease activity outcome measure for pregnant SLE patients
Source: Rheumatol Adv Pract. 2022 Oct 3;6(3):rkac081. doi: 10.1093/rap/rkac081 (PMC9585949; doi:10.1093/rap/rkac081)
Supplement: rkac081_Supplementary_Data [file rkac081_supplementary_data.zip › Supplementary Data S2. BILAG2004-P_Glossary.doc]

# BILAG2004-Pregnancy INDEX GLOSSARY

INSTRUCTIONS

• to be used during pregnancy and post-partum period

• only record features that are **attributable to SLE disease activity** **and not due to**

**pathophysiological changes of pregnancy, damage, infection, thrombosis (in**

**absence of inflammatory process) or other conditions**

• assessment refers to manifestations occurring in the **last 4 weeks compared with the**

**previous 4 weeks**

• activity refers to disease process which is reversible while damage refers to permanent

process/scarring (irreversible)

• damage due to SLE should be considered as a cause of features that are fixed/persistent

(SLICC/ACR damage index uses persistence ≥ 6 months to define damage)

• in some manifestations, it may be difficult to differentiate SLE from other conditions as

there may not be any specific test and the decision would then lies with the **physician’s**

**judgement on the balance of probabilities**

• ophthalmic manifestations would usually need to be assessed by ophthalmologist (using

the proforma included with this glossary) and these items would need to be recorded

retrospectively after receiving the response from ophthalmologist

• guidance for scoring:

**(4) New**

• manifestations are recorded as new when it is a new episode occurring in the last

4 weeks (compared to the previous 4 weeks) which has not improved and this

includes new episodes (recurrence) of old manifestations

• new episode occurring in the last 4 weeks but also satisfying the criteria for

improvement (below) would be classified as improving instead of new

**(3) Worse**

• this refers to manifestations that have deteriorated/worsened significantly in the

last 4 weeks compared to the previous 4 weeks, sufficient for consideration of

increase in therapy

**(2) Same**

• this refers to manifestations that have been present for the last 4 weeks and the

previous 4 weeks without significant improvement or deterioration (from the

previous 4 weeks)

• this also applies to manifestations that have improved over the last 4 weeks

compared to the previous 4 weeks but do not meet the criteria for improvement

**(1) Improving**

• definition of **improvement**: (a) the amount of improvement is sufficient for

**consideration of reduction in therapy** and

would not justify escalation in therapy

**AND**

(b) improvement must be **present currently and**

**for at least 2 weeks** out of the last 4 weeks

**OR**

manifestation that has **completely resolved** **and**

**remained absent** at least over the **whole of last 1**

**week**

**(0) Not present**

**(ND) Not done**

• it is important to indicate if a test has not been performed (particularly laboratory

investigations) so that this will be recorded as such in the database & not as

normal or absent (which is the default)

**Y/N - Indicate (tick) if not due to SlE activity**

• for descriptors that are based on measurements (in renal and haematology

systems), it is important to indicate if these are not due to lupus disease activity

(for consideration of scoring) as they are usually recorded routinely into a

database

**TRICKLE DOWN RULE**

• when item of highest level of severity is recorded, similar item of lower level of

severity must be recorded as well (item of lower level of severity must not be

recorded as not present)

**CHANGE IN SEVERITY CATEGORY**

• there are several items in the index which have been categorised into mild and

severe (depending on definition)

• if a mild item deteriorated to the extent that it fulfilled the definition of severe

category (ie changed into severe category) within the last 4 weeks:

severe item should be scored as new (4)

**AND** mild item scored as worsening (3)

• if a severe item improved (fulfilling the improvement criteria) to the extent that it

no longer fulfilled the definition of severe category (ie changed into mild

category) within the last 4 weeks:

severe item scored as not present (0) if criteria for severe category has not

been met over last 4 weeks

**or** as improving (1) if criteria for severe category has been

met at some point over last 4 weeks

**AND**

mild item scored as improving (1) if it is improving over last 4 weeks

**or** as the same (2) if it has remained stable over last 4 weeks

# RECORDING ALGORITHM

**Period B – 4 weeks**

**Period A – 4 weeks**

**Assessment**

**Manifestation over last 4 weeks (Period A) ?**

***No***

***Not present (0)***

***Yes***

**Is it due to SLE disease activity ?**

***No***

***Not present (0)***

***Yes***

**Compared to previous 4 weeks (Period B), is it new (not present in Period B) ?**

***Note Trickle Down Rule***

***Improved (1)***

***or***

***Same (2)***

***or***

***Worse (3)***

**Has it improved over last 4 weeks (Period A) ?**

***Note improvement criteria***

***No***

***No***

***New (4)***

***Improved (1)***

***Yes***

***Yes***

**CONSTITUTIONAL**

1. Pyrexia	temperature > 37.5˚C documented

2. Unintentional weight loss > 5%

3. Lymphadenopathy	palpable lymph node more than 1 cm diameter

exclude infection

4. Anorexia

# MUCOCUTANEOUS

5. Severe eruption	> 18% body surface area

any lupus rash except panniculitis, bullous lesion

& angio-oedema

body surface area (BSA) is estimated using the rules of nines (used to assess extent of burns) as follows:

palm(excluding fingers) = 1% BSA

       	each lower limb = 18% BSA

       	each upper limb = 9% BSA

       	torso (front) = 18% BSA

       	torso (back) = 18% BSA

       	head = 9% BSA

       	genital (male) = 1% BSA

6. Mild eruption	**≤** 18% body surface area

any lupus rash except panniculitis, bullous lesion

& angio-oedema

malar rash must have been observed by a

physician and has to be present continuously

(persistent) for at least 1 week to be considered

significant (to be recorded)

exclude transient facial blush of pregnancy and melasma/chloasma

7. Severe angio-oedema	potentially life-threatening eg: stridor

angio-oedema is a variant form of urticaria

which affects the subcutaneous, submucosal and

deep dermal tissues

8. Mild angio-oedema	not life threatening

9. Severe mucosal ulceration	disabling (significantly interfering with oral

intake), extensive & deep ulceration

must have been observed by a physician

10. Mild mucosal ulceration	localised &/or non-disabling ulceration

11. Severe panniculitis or bullous lupus	any one:

> 9% body surface area

facial panniculitis

panniculitis that is beginning to ulcerate

panniculitis that threatens integrity of

subcutaneous tissue (beginning to cause

surface depression) on > 9% body surface

area

panniculitis presents as a palpable and tender

subcutaneous induration/nodule

note that established surface depression and

atrophy alone is likely to be due to damage

12. Mild panniculitis or bullous lupus	≤ 9% body surface area

does not fulfil any criteria for severe panniculitis (for panniculitis)

13. Major cutaneous vasculitis/thrombosis	resulting in extensive gangrene or ulceration or

skin infarction

14. Digital infarct or nodular vasculitis	localised single or multiple infarct(s) over

digit(s) or tender erythematous nodule(s)

15. Severe alopecia	clinically detectable (diffuse or patchy) hair loss

with scalp inflammation (redness over scalp)

16. Mild alopecia	diffuse or patchy hair loss without scalp inflammation (clinically detectable or by history)

exclude post-partum alopecia

17. Peri-ungual erythema or chilblains	chilblains are localised inflammatory lesions

(may ulcerate) which are precipitated by

exposure to cold

18. Splinter haemorrhages

# NEUROPSYCHIATRIC

19. Aseptic meningitis	criteria (all): acute/subacute onset

headache

fever

abnormal CSF (raised protein &/or

lymphocyte predominance) but negative

cultures

preferably photophobia, neck stiffness and

meningeal irritation should be present as well but

are not essential for diagnosis

exclude CNS/meningeal infection, intracranial

haemorrhage

20. Cerebral vasculitis	should be present with features of vasculitis

in another system

supportive imaging &/or biopsy findings

21. Demyelinating syndrome	discrete white matter lesion with associated

neurological deficit not recorded elsewhere

ideally there should have been at least one previously recorded event

supportive imaging required

exclude multiple sclerosis

22. Myelopathy	acute onset of rapidly evolving paraparesis or

quadriparesis and/or sensory level

exclude intramedullary and extramedullary

space occupying lesion

23. Acute confusional state	acute disturbance of consciousness or level of

arousal with reduced ability to focus, maintain or shift attention

includes hypo- and hyperaroused states and encompasses the spectrum from delirium to coma

24. Psychosis	delusion or hallucinations

does not occur exclusively during course of a

delirium

exclude post-partum psychosis, drugs, substance

abuse, primary psychotic disorder

25. Acute inflammatory demyelinating	criteria:

polyradiculoneuropathy	progressive polyradiculoneuropathy

loss of reflexes

symmetrical involvement

increased CSF protein without pleocytosis

supportive electrophysiology study

26. Mononeuropathy (single/multiplex)	supportive electrophysiology study required

exclude carpal tunnel syndrome associated with

pregnancy

27. Cranial neuropathy	except optic neuropathy which is classified

under ophthalmic system

28. Plexopathy	disorder of brachial or lumbosacral plexus

resulting in neurological deficit not

corresponding to territory of single root or nerve

supportive electrophysiology study required

29. Polyneuropathy	acute symmetrical distal sensory and/or motor

deficit

supportive electrophysiology study required

30. Seizure disorder	independent description of seizure by reliable witness

exclude eclampsia

31. Status epilepticus	a seizure or series of seizures lasting ≥ 30

minutes without full recovery to baseline

exclude eclampsia

32. Cerebrovascular disease	any one with supporting imaging:

(not due to vasculitis)	stroke syndrome

transient ischaemic attack

intracranial haemorrhage

exclude eclampsia, hypoglycaemia, cerebral sinus thrombosis, vascular malformation, tumour, abscess

cerebral sinus thrombosis not included as

definite thrombosis not considered part of lupus activity

33. Cognitive dysfunction	significant deficits in any cognitive functions:

simple attention (ability to register & maintain

information)

complex attention

memory (ability to register, recall & recognise

information eg learning, recall)

visual-spatial processing (ability to analyse,

synthesise & manipulate visual-spatial

information)

language (ability to comprehend, repeat &

produce oral/written material eg verbal

fluency, naming)

reasoning/problem solving (ability to reason &

abstract)

psychomotor speed

executive functions (eg planning, organising,

sequencing)

in absence of disturbance of consciousness or

level of arousal

sufficiently severe to interfere with daily

activities

neuropsychological testing should be done or

corroborating history from third party if possible

exclude substance abuse

34. Movement disorder	exclude drugs

35. Autonomic disorder	any one:

fall in blood pressure to standing > 30/15 mm

Hg (systolic/diastolic)

increase in heart rate to standing ≥ 30 bpm

loss of heart rate variation with respiration

(max – min < 15 bpm, expiration:inspiration

ratio < 1.2, Valsalva ratio < 1.4)

loss of sweating over body and limbs

(anhidrosis) by sweat test

exclude drugs and diabetes mellitus

36. Cerebellar ataxia	cerebellar ataxia in isolation of other CNS features

usually subacute presentation

37. Severe lupus headache (unremitting)	disabling headache unresponsive to narcotic analgesia & lasting ≥ 3 days

exclude intracranial space occupying lesion

and CNS infection

38. Headache from IC hypertension	exclude cerebral sinus thrombosis

# MUSCULOSKELETAL

39. Severe myositis	significantly elevated serum muscle enzymes

with significant muscle weakness

exclude endocrine causes and drug-induced

myopathy

electromyography and muscle biopsy are used for diagnostic purpose and are not required to determine level of activity

40. Mild myositis	significantly elevated serum muscle enzymes

with myalgia but without significant muscle

weakness

asymptomatic elevated serum muscle enzymes

not included

exclude endocrine causes and drug-induced

myopathy

electromyography and muscle biopsy are used for diagnostic purpose and are not required to determine level of activity

41. Severe arthritis	observed active synovitis ≥ 2 joints with marked

loss of functional range of movements and

significant impairment of activities of daily

living, that has been present on several days

(cumulatively) over the last 4 weeks

42. Moderate Arthritis or Tendonitis tendonitis/tenosynovitis or active synovitis ≥ 1

or Tenosynovitis	joint (observed or through history) with some loss of functional range of movements, that has been present on several days over the last 4 weeks

exclude bland effusion of knee and peripheral

oedema of pregnancy

43. Mild Arthritis or Arthralgia or Myalgia	inflammatory type of pain (worse in the morning with stiffness, usually improves with activity & not brought on by activity) over joints/muscle

inflammatory arthritis which does not fulfil the above criteria for moderate or severe arthritis

exclude bland effusion of knee, peripheral

oedema of pregnancy and mechanical pain of

pregnancy

# CARDIORESPIRATORY

44. Mild myocarditis	inflammation of myocardium with raised

cardiac enzymes &/or ECG changes and without resulting cardiac failure, arrhythmia or valvular dysfunction

45. Cardiac failure	cardiac failure due to myocarditis or non-infective inflammation of endocardium or cardiac valves (endocarditis)

cardiac failure due to myocarditis is defined by left ventricular ejection fraction ≤ 40% & pulmonary oedema or peripheral oedema

cardiac failure due to acute valvular regurgitation (from endocarditis) can be associated with normal left ventricular ejection fraction

diastolic heart failure is not included

46. Arrhythmia	arrhythmia (except sinus tachycardia) due to myocarditis or non-infective inflammation of endocardium or cardiac valves (endocarditis)

confirmation by electrocardiogram required

(history of palpitations alone inadequate)

47. New valvular dysfunction	new cardiac valvular dysfunction due to myocarditis or non-infective inflammation of endocardium or cardiac valves (endocarditis)

supportive imaging required

48. Pleurisy/Pericarditis	convincing history &/or physical findings that you would consider treating

in absence of cardiac tamponade or pleural effusion with dyspnoea

do not score if you are unsure whether or not it is pleurisy/pericarditis

49. Cardiac tamponade	supportive imaging required

50. Pleural effusion with dyspnoea	supportive imaging required

51. Pulmonary haemorrhage/vasculitis	inflammation of pulmonary vasculature with

haemoptysis &/or dyspnoea &/or pulmonary hypertension

supportive imaging &/or histological diagnosis required

52. Interstitial alveolitis/pneumonitis	radiological features of alveolar infiltration not

due to infection or haemorrhage required for diagnosis

corrected gas transfer Kco reduced to < 70% normal or fall of > 20% if previously abnormal

on-going activity would be determined by

clinical findings and lung function tests, and

repeated imaging may be required in those with

deterioration (clinically or lung function tests) or failure to respond to therapy

53. Shrinking lung syndrome	acute reduction (> 20% if previous measurement

available) in lung volumes (to < 70% predicted)

in the presence of normal corrected gas transfer

(Kco) & dysfunctional diaphragmatic

movements

54. Aortitis	inflammation of aorta (with or without

dissection) with supportive imaging abnormalities

accompanied by > 10 mm Hg difference in BP between arms &/or claudication of extremities &/or vascular bruits

repeated imaging would be required to determine

on-going activity in those with clinical

deterioration or failure to respond to therapy

55. Coronary vasculitis	inflammation of coronary vessels with

radiographic evidence of non-atheromatous narrowing, obstruction or aneurysmal changes

# GASTROINTESTINAL

56. Lupus peritonitis	serositis presenting as acute abdomen with

rebound/guarding

57. Serositis	not presenting as acute abdomen

58. Lupus enteritis or colitis	vasculitis or inflammation of small or large bowel with supportive imaging &/or biopsy findings

59. Malabsorption	diarrhoea with abnormal D- xylose absorption

test or increased faecal fat excretion after exclusion of coeliac’s disease (poor response to gluten-free diet) and gut vasculitis

60. Protein-losing enteropathy	diarrhoea with hypoalbuminaemia or increased

faecal excretion of iv radiolabeled albumin after exclusion of gut vasculitis and malabsorption

61. Intestinal pseudo-obstruction	subacute intestinal obstruction due to intestinal

hypomotility

62. Lupus hepatitis	raised transaminases

absence of autoantibodies specific to autoimmune hepatitis (eg: anti-smooth muscle, anti-liver cytosol 1) &/or biopsy appearance of chronic active hepatitis

hepatitis typically lobular with no piecemeal necrosis

exclude HELLP syndrome

63. Acute lupus cholecystitis	after exclusion of gallstones and infection

64. Acute lupus pancreatitis	usually associated multisystem involvement

# OPHTHALMIC

65. Orbital inflammation	orbital inflammation with myositis &/or extra-

ocular muscle swelling &/or proptosis

supportive imaging required

66. Severe keratitis	sight threatening

includes: corneal melt

peripheral ulcerative keratitis

67. Mild keratitis	not sight threatening

68. Anterior uveitis

69. Severe posterior uveitis &/or retinal	sight-threatening &/or retinal vasculitis

vasculitis	not due to vaso-occlusive disease

70. Mild posterior uveitis &/or retinal	not sight-threatening

vasculitis

not due to vaso-occlusive disease

71. Episcleritis

72. Severe scleritis	necrotising anterior scleritis

anterior &/or posterior scleritis requiring

systemic steroids/immunosuppression &/or not responding to NSAIDs

73. Mild scleritis	anterior &/or posterior scleritis not requiring systemic steroids

excludes necrotising anterior scleritis

74. Retinal/choroidal vaso-occlusive	includes: retinal arterial & venous occlusion

disease	serous retinal &/or retinal pigment

epithelial detachments secondary to

choroidal vasculopathy

75. Isolated cotton-wool spots	also known as cytoid bodies

76. Optic neuritis	excludes anterior ischaemic optic neuropathy

77. Anterior ischaemic optic neuropathy	visual loss with pale swollen optic disc due to occlusion of posterior ciliary arteries

# RENAL

78. Complement C3

79. Complement C4

80. Anti-dsDNA

81. Urine dipstick	exclude pre-eclampsia/eclampsia (proteinuria after 20 weeks gestation in the setting of rising BP and absence of active urinary sediment is most likely due to pre-eclampsia)

82. Urine albumin-creatinine ratio	on freshly voided urine sample

exclude pre-eclampsia/eclampsia (proteinuria after 20 weeks gestation in the setting of rising BP and absence of active urinary sediment is most likely due to pre-eclampsia)

rise of > 50% before 20 weeks gestation in those with pre-existing damage-related proteinuria suggests presence of superimposed pathology

conversion: 1 mg/mg = 113 mg/mmol

83. Urine protein-creatinine ratio	on freshly voided urine sample

exclude pre-eclampsia/eclampsia (proteinuria after 20 weeks gestation in the setting of rising BP and absence of active urinary sediment is most likely due to pre-eclampsia)

rise of > 50% before 20 weeks gestation in those with pre-existing damage-related proteinuria suggests presence of superimposed pathology

conversion: 1 mg/mg = 113 mg/mmol

84. 24 hour urine protein	exclude pre-eclampsia/eclampsia (proteinuria after 20 weeks gestation in the setting of rising BP and absence of active urinary sediment is most likely due to pre-eclampsia)

rise of > 50% before 20 weeks gestation in those with pre-existing damage-related proteinuria suggests presence of superimposed pathology

85. Nephrotic syndrome	criteria:

heavy proteinuria (> 50 mg/kg/day or >

3.5 g/day or protein-creatinine ratio > 350

mg/mmol or albumin-creatinine ratio > 350

mg/mmol)

hypoalbuminaemia

oedema

86. Plasma/Serum creatinine

87. Creatinine clearance

88. Active urinary sediment	pyuria (> 5 WCC/hpf or > 10 WCC/mm^3^ (μl))

OR

haematuria (> 5 RBC/hpf or > 10 RBC/mm^3^ (μl))

OR

red cell casts

OR

white cell casts

in absence of other causes (especially infection,

vaginal bleed, calculi)

89. Histology of active nephritis	WHO Classification (1995): (any one)

Class III – (a) or (b) subtypes

Class IV – (a), (b) or (c) subtypes

Class V – (a), (b), (c) or (d) subtypes

Vasculitis

OR

ISN/RPS Classification (2003): (any one)

Class III – (A) or (A/C) subtypes

Class IV – (A) or (A/C) subtypes

Class V

Vasculitis

within last 3 months

glomerular sclerosis without inflammation not included

# HAEMATOLOGY

90. Haemoglobin	exclude dietary deficiency & GI blood loss

91. White cell count

92. Neutrophil count

93. Lymphocyte count

94. Platelet count	exclude thrombocytopenia of pre-eclampsia,

HELLP syndrome & antiphospholipid syndrome

95. TTP	thrombotic thrombocytopaenic purpura

clinical syndrome of micro-angiopathic haemolytic anaemia and thrombocytopenia in absence of any other identifiable cause

96. Evidence of active haemolysis	positive Coombs’ test & evidence of haemolysis (raised bilirubin or raised reticulocyte count or reduced haptoglobulins or fragmented RBC or microspherocytes)

exclude HELLP syndrome

97. Isolated positive Coombs’ test

# ADDITIONAL ITEMS

i.	Weight

ii.	Blood pressure

iii.	Expected date of delivery

iv. Gestation in weeks

v. Postpartum period in weeks
