# Supplementary material for: The BILAG2004-Pregnancy Index is a valid disease activity outcome measure for pregnant SLE patients
Source: Rheumatol Adv Pract. 2022 Oct 3;6(3):rkac081. doi: 10.1093/rap/rkac081 (PMC9585949; doi:10.1093/rap/rkac081)
Supplement: rkac081_Supplementary_Data [file rkac081_supplementary_data.zip › Supplementary Data S3. BILAG2004-P_Scoring.doc]

**BILAG2004-Pregnancy INDEX SCORING**

• scoring based on the principle of physician’s intention to treat

| **Category** | **Definition** |
| --- | --- |
| **A** | **Severe disease activity requiring any of the following treatment:**  **1. systemic high dose oral glucocorticoids (equivalent to prednisolone > 20**  **mg/day)**  **2. intravenous pulse glucocorticoids (equivalent to pulse methylprednisolone**  **≥ 500 mg)**  **3. systemic immunomodulators (include biologicals, immunoglobulins and**  **plasmapheresis)**  **4. therapeutic high dose anticoagulation in the presence of high dose steroids**  **or immunomodulators** |
| **B** | **Moderate disease activity requiring any of the following treatment:**  **1. systemic low dose oral glucocorticoids (equivalent to prednisolone ≤ 20**  **mg/day)**  **2. intramuscular or intra-articular or soft tissue glucocorticoids injection**  **(equivalent to methylprednisolone < 500mg)**  **3. topical glucocorticoids**  **4. topical immunomodulators**  **5. antimalarials**  **6. symptomatic therapy**  **eg: NSAIDs for inflammatory arthritis** |
| **C** | **Stable mild disease** |
| **D** | **Inactive disease but previously affected** |
| **E** | **System never involved** |

**CONSTITUTIONAL**

**Category A:**

Pyrexia recorded as 2 (same), 3 (worse) or 4 (new) **AND**

Any 2 or more of the following recorded as 2 (same), 3 (worse) or 4 (new):

Weight loss

Lymphadenopathy/splenomegaly

Anorexia

**Category B:**

Pyrexia recorded as 2 (same), 3 (worse) or 4 (new) **OR**

Any 2 or more of the following recorded as 2 (same), 3 (worse) or 4 (new):

Weight loss

Lymphadenopathy/splenomegaly

Anorexia

**BUT** do not fulfil criteria for Category A

**Category C**

Pyrexia recorded as 1 (improving) **OR**

One or more of the following recorded as > 0:

Weight loss

Lymphadenopathy/Splenomegaly

Anorexia

**BUT** does not fulfil criteria for category A or B

**Category D**

Previous involvement

**Category E**

No previous involvement

##### MUCOCUTANEOUS

**Category A**

Any of the following recorded as 2 (same), 3 (worse) or 4 (new):

Skin eruption - severe

Angio-oedema - severe

Mucosal ulceration - severe

Panniculitis/Bullous lupus - severe

Major cutaneous vasculitis/thrombosis

**Category B**

Any Category A features recorded as 1 (improving) **OR**

Any of the following recorded as 2 (same), 3 (worse) or 4 (new):

Skin eruption - mild

Panniculitis/Bullous lupus - mild

Digital infarcts or nodular vasculitis

Alopecia - severe

**Category C**

Any Category B features recorded as 1 (improving) **OR**

Any of the following recorded as > 0:

Angio-oedema - mild

Mucosal ulceration - mild

Alopecia - mild

Periungual erythema/chilblains

Splinter haemorrhages

**Category D**

Previous involvement

**Category E**

No previous involvement

# NEUROPSYCHIATRIC

# Category A

Any of the following recorded as 2 (same), 3 (worse) or 4 (new):

Aseptic meningitis

Cerebral vasculitis

Demyelinating syndrome

Myelopathy

Acute confusional state

Psychosis

Acute inflammatory demyelinating polyradiculoneuropathy

Mononeuropathy (single/multiplex)

Cranial neuropathy

Plexopathy

Polyneuropathy

Status epilepticus

Cerebellar ataxia

# Category B

Any Category A features recorded as 1 (improving) **OR**

Any of the following recorded as 2 (same), 3 (worse) or 4 (new):

Seizure disorder

Cerebrovascular disease (not due to vasculitis)

Cognitive dysfunction

Movement disorder

Autonomic disorder

Headache severe unremitting

Headache due to raised intracranial hypertension

**Category C**

Any Category B features recorded as 1 (improving)

**Category D**

Previous involvement

**Category E**

No previous involvement

**MUSCULOSKELETAL**

# Category A

Any of the following recorded as 2 (same), 3 (worse) or 4 (new):

Severe Myositis

Severe arthritis

**Category B**

Any Category A features recorded as 1 (improving) **OR**

Any of the following recorded as 2 (same), 3 (worse) or 4 (new):

Mild Myositis

Arthritis(moderate)/Tendonitis/Tenosynovitis

# Category C

Any Category B features recorded as 1 (improving) **OR**

Any of the following recorded as > 0:

Arthritis(mild)/Arthralgia/Myalgia

# Category D

Previous involvement

**Category E**

No previous involvement

**CARDIORESPIRATORY**

**Category A**

Any of the following recorded as 2 (same), 3 (worse) or 4 (new):

Myocarditis/Endocarditis + Cardiac failure

Arrhythmia

New valvular dysfunction

Cardiac tamponade

Pleural effusion with dyspnoea

Pulmonary haemorrhage/vasculitis

Interstitial alveolitis/pneumonitis

Shrinking lung syndrome

Aortitis

Coronary vasculitis

**Category B**

Any Category A features recorded as 1 (improving) **OR**

Any of the following recorded as 2 (same), 3 (worse) or 4 (new):

Pleurisy/Pericarditis

Myocarditis - mild

**Category C**

Any Category B features recorded as 1 (improving)

**Category D**

Previous involvement

**Category E**

No previous involvement

**GASTROINTESTINAL**

# Category A

Any of the following recorded as 2 (same), 3 (worse) or 4 (new):

Peritonitis

Lupus enteritis/colitis

Intestinal pseudo-obstruction

Acute lupus cholecystitis

Acute lupus pancreatitis

# Category B

Any Category A feature recorded as 1 (improving) **OR**

Any of the following recorded as 2 (same), 3 (worse) or 4 (new):

Abdominal serositis and/or ascites

Malabsorption

Protein losing enteropathy

Lupus hepatitis

# Category C

Any Category B features recorded as 1 (improving)

**Category D**

Previous involvement

**Category E**

No previous involvement

**OPHTHALMIC**

# Category A

Any of the following recorded as 2 (same), 3 (worse) or 4 (new):

Orbital inflammation/myositis/proptosis

Keratitis - severe

Posterior uveitis/retinal vasculitis - severe

Scleritis - severe

Retinal/choroidal vaso-occlusive disease

Optic neuritis

Anterior ischaemic optic neuropathy

# Category B

Any Category A features recorded as 1 (improving) **OR**

Any of the following recorded as 2 (same), 3 (worse) or 4 (new):

Keratitis - mild

Anterior uveitis

Posterior uveitis/retinal vasculitis - mild

Scleritis - mild

# Category C

Any Category B features recorded as 1 (improving) **OR**

Any of the following recorded as > 0:

Episcleritis

Isolated cotton-wool spots (cytoid bodies)

# Category D

Previous involvement

# Category E

No previous involvement

**RENAL**

# Category A

**Two or more** of the following **providing 1, 3 or 4 is included**:

1. (i) Deteriorating proteinuria (severe) defined as

(a) urine dipstick increased by ≥ 2 levels (used only if other methods of urine protein estimation

not available); **or**

(b) 24 hour urine protein > 1 g that has not decreased (improved) by ≥ 25%; **or**

(c) urine protein-creatinine ratio > 100 mg/mmol that has not decreased (improved) by ≥ 25%;

**or**

(d) urine albumin-creatinine ratio > 100 mg/mmol that has not decreased (improved) by ≥ 25%;

**AND**

(ii) Abnormal immunological tests

(a) C3 or C4 decrease by ≥ 10%; **or**

(b) anti-dsDNA level above normal range and rise by ≥ 25%

2. Deteriorating renal function (severe) defined as

(a) plasma creatinine > 70 μmol/l and having risen to > 120% of previous value; **or**

(b) creatinine clearance having fallen to < 80% of previous value; **or**

(c) creatinine clearance < 100 ml/min per 1.73 m^2^, and last time was > 100 ml/min per 1.73 m^2^ or

was not measured.

3. Active urinary sediment

4. Histological evidence of active nephritis within last 3 months

5. Nephrotic syndrome

# Category B

One of the following but has not fulfilled criteria for Category A:

1. One of Category A feature

2. Deteriorating proteinuria (without immunological changes) defined as

(a) urine dipstick which has risen by 1 level to at least 2+ (used only if other methods of urine

protein estimation not available); **or**

(b) 24 hour urine protein > 1 g that has not decreased (improved) by ≥ 25%; **or**

(c) urine protein-creatinine ratio > 100 mg/mmol that has not decreased (improved) by ≥ 25%; **or**

(d) urine albumin-creatinine ratio > 100 mg/mmol that has not decreased (improved) by ≥ 25%

3. Plasma creatinine > 70 μmol/l and having risen to ≥ 110% but ≤ 120% of previous value

# Category C

Mild proteinuria defined as

(a) urine dipstick ≥ 1+ but has not fulfilled criteria for Category A & B (used only if other methods

of urine protein estimation not available); **or**

(b) 24 hour urine protein > 0.50 g but has not fulfilled criteria for Category A & B ; **or**

(c) urine protein-creatinine ratio > 50 mg/mmol but has not fulfilled criteria for Category A & B; **or**

(d) urine albumin-creatinine ratio > 50 mg/mmol but has not fulfilled criteria for Category A & B

# Category D

Previous involvement

# Category E

No previous involvement

Note: although albumin-creatinine ratio and protein-creatinine ratio are different, we use the same cut-

off values for this index

**HAEMATOLOGY**

**Category A**

TTP recorded as 2 (same), 3 (worse) or 4 (new)	**OR**

Any of the following:

Evidence of haemolysis and Haemoglobin < 8 g/dl

Platelet count	< 25 x 10^9^/l

# Category B

TTP recorded as 1 (improving)	**OR**

Any of the following:

Evidence of haemolysis and Haemoglobin 8 - 8.9 g/dl

Haemoglobin	< 8 g/dl (without haemolysis)

White cell count	< 1.0 x 10^9^/l

Neutrophil count	< 0.5 x 10^9^/l

Platelet count	25 - 49 x 10^9^/l

# Category C

Any of the following:

Evidence of haemolysis and Haemoglobin ≥ 9 g/dl

Haemoglobin	8 - 9.9 g/dl (without haemolysis)

White cell count	1 - 3.9 x 10^9^/l

Neutrophil count	0.5 - 1.9 x 10^9^/l

Lymphocyte count	< 1.0 x 10^9^/L

Platelet count	50 - 149 x 10^9^/l

Isolated Coombs’ test positive

# Category D

Previous involvement

# Category E

No previous involvement
