# Supplementary material for: The BILAG2004-Pregnancy Index is a valid disease activity outcome measure for pregnant SLE patients
Source: Rheumatol Adv Pract. 2022 Oct 3;6(3):rkac081. doi: 10.1093/rap/rkac081 (PMC9585949; doi:10.1093/rap/rkac081)
Supplement: rkac081_Supplementary_Data [file rkac081_supplementary_data.zip › Supplementary Data S1. BILAG2004-P_Form.doc]

♦ Only record manifestations/items due to SLE Disease Activity

♦ Assessment refers to manifestations occurring in the last 4 weeks (compared with the previous 4 weeks)

♦ TO BE USED WITH THE GLOSSARY

Record: **ND	Not Done**

**0	Not present**

**1	Improving**

**2	Same**

###	3	Worse

**4	New**

**Yes/No OR Value	(where indicated)**

***Y/N**	**Confirm this is due to SLE activity (Yes/No)**

# CONSTITUTIONAL

1. Pyrexia - documented > 37.5ºC	(	)

2. Weight loss - unintentional > 5%	(	)

3. Lymphadenopathy/splenomegaly	(	)

4. Anorexia	(	)

# MUCOCUTANEOUS

5. Skin eruption - severe	(	)

6. Skin eruption - mild	(	)

7. Angio-oedema - severe	(	)

8. Angio-oedema - mild	(	)

9. Mucosal ulceration - severe	(	)

10. Mucosal ulceration - mild	(	)

11. Panniculitis/Bullous lupus - severe	(	)

12. Panniculitis/Bullous lupus - mild	(	)

13. Major cutaneous vasculitis/thrombosis	(	)

14. Digital infarcts or nodular vasculitis	(	)

15. Alopecia - severe	(	)

16. Alopecia - mild	(	)

17. Peri-ungual erythema/chilblains	(	)

18. Splinter haemorrhages	(	)

# NEUROPSYCHIATRIC

19. Aseptic meningitis	(	)

20. Cerebral vasculitis	(	)

21. Demyelinating syndrome	(	)

22. Myelopathy	(	)

23. Acute confusional state	(	)

24. Psychosis	(	)

25. Acute inflammatory demyelinating	(	)

polyradiculoneuropathy

26. Mononeuropathy (single/multiplex)	(	)

27. Cranial neuropathy	(	)

28. Plexopathy	(	)

29. Polyneuropathy	(	)

30. Seizure disorder	(	)

31. Status epilepticus	(	)

32. Cerebrovascular disease (not due to	vasculitis)	(	)

33. Cognitive dysfunction	(	)

34. Movement disorder	(	)

35. Autonomic disorder	(	)

36. Cerebellar ataxia (isolated)	(	)

37. Lupus headache - severe unremitting	(	)

38. Headache from IC hypertension	(	)

# MUSCULOSKELETAL

39. Myositis - severe	(	)

40. Myositis - mild	(	)

41. Arthritis (severe)	(	)

42. Arthritis (moderate)/Tendonitis/Tenosynovitis	(	)

43. Arthritis (mild)/Arthralgia/Myalgia	(	)

**Weight (kg):	BP (mm Hg):**

**EDD (date):	Gestation (weeks):**

**Delivery (date):	Postpartum (weeks):**

# CARDIORESPIRATORY

44. Myocarditis - mild	(	)

45. Myocarditis/Endocarditis + Cardiac failure	(	)

46. Arrhythmia	(	)

47. New valvular dysfunction	(	)

48. Pleurisy/Pericarditis	(	)

49. Cardiac tamponade	(	)

50. Pleural effusion with dyspnoea	(	)

51. Pulmonary haemorrhage/vasculitis	(	)

52. Interstitial alveolitis/pneumonitis	(	)

53. Shrinking lung syndrome	(	)

54. Aortitis	(	)

55. Coronary vasculitis	(	)

# GASTROINTESTINAL

56. Lupus peritonitis	(	)

57. Abdominal serositis or ascites	(	)

58. Lupus enteritis/colitis	(	)

59. Malabsorption	(	)

60. Protein losing enteropathy	(	)

61. Intestinal pseudo-obstruction	(	)

62. Lupus hepatitis	(	)

63. Acute lupus cholecystitis	(	)

64. Acute lupus pancreatitis	(	)

# OPHTHALMIC

65. Orbital inflammation/myositis/proptosis	(	)

66. Keratitis - severe	(	)

67. Keratitis - mild	(	)

68. Anterior uveitis	(	)

69. Posterior uveitis/retinal vasculitis - severe	(	)

70. Posterior uveitis/retinal vasculitis - mild	(	)

71. Episcleritis	(	)

72. Scleritis - severe	(	)

73. Scleritis - mild	(	)

74. Retinal/choroidal vaso-occlusive disease	(	)

75. Isolated cotton-wool spots (cytoid bodies)	(	)

76. Optic neuritis	(	)

77. Anterior ischaemic optic neuropathy	(	)

# RENAL

78. Complement C3	value	(	)

79. Complement C4	value	(	)

80. Anti-dsDNA	value	(	)

81. Urine dipstick protein	(+=1, ++=2, +++=3)	(	) **Y/N***

82. Urine albumin-creatinine ratio	mg/mmol	(	) **Y/N***

83. Urine protein-creatinine ratio	mg/mmol	(	) **Y/N***

84. 24 hour urine protein (g)	value	(	) **Y/N***

85. Nephrotic syndrome	Yes/No	(	)

86. Creatinine (plasma/serum)	μmol/l	(	) **Y/N***

87. Creatinine clearance	ml/min/1.73 m^2^ (	) **Y/N***

88. Active urinary sediment	Yes/No	(	)

89. Active nephritis	Yes/No	(	)

# HAEMATOLOGY

90. Haemoglobin (g/dl)	value	(	) **Y/N***

91. Total white cell count (x 10^9^/l)	value	(	) **Y/N***

92. Neutrophils (x 10^9^/l)	value	(	) **Y/N***

93. Lymphocytes (x 10^9^/l)	value	(	) **Y/N***

94. Platelets (x 10^9^/l)	value	(	) **Y/N***

95. TTP	(	)

96. Evidence of active haemolysis	Yes/No	(	)

97. Coombs’ test positive (isolated)	Yes/No	(	)
